# Cutaneous Squamous Cell Carcinoma Mimickers: Beware the Pruritic Papule on the Leg of an Older Female

**DOI:** 10.7759/cureus.41569

**Published:** 2023-07-08

**Authors:** Shelby L Kubicki, Timothy J Hansen, Deborah F MacFarlane

**Affiliations:** 1 Dermatology, The University of Texas Health Science Center at Houston (UTHealth), Houston, USA; 2 Dermatology, McFarland Clinic, Ames, USA; 3 Dermatology, The University of Texas MD Anderson Cancer Center, Houston, USA

**Keywords:** lichen planus, female, pseudoepitheliomatous hyperplasia, pruritic papule, mimickers, squamous cells carcinoma

## Abstract

Background

Correctly identifying cutaneous squamous cell carcinoma (cSCC) mimickers can be both clinically and histopathologically challenging. We present a series of patients with biopsy-proven cSCCs for whom multiple surgeries were avoided by assessing the clinical situation, recognizing an alternative diagnosis that pathologically mimics cSCC, and prescribing appropriate therapy for the underlying condition.

Methodology

Seven female patients presented for treatment of biopsy-proven cSCC affecting the lower leg. After further investigation, we observed that these women shared similar characteristics, including age ranging from the 5th to the 9th decade, often experiencing stress, exhibiting associated pruritus with diverse causes, having few or no previous skin cancers, and generally showing minimal photodamage.

Results

In all cases, surgery was deferred and patients demonstrated complete clinical response to therapies directed toward an alternative diagnosis. Repeat biopsies of treated lesions revealed no evidence of cSCC.

Conclusions

Not all histologically diagnosed cases of cSCC should be treated with surgery, and each patient should be worked up thoroughly to evaluate for an alternative diagnosis. Possible clinical and histologic cSCC mimickers include allergic contact dermatitis (ACD), stasis dermatitis, hypertrophic lichen planus (LP), and drug eruption, among others. In the described population, pruritic papules on the lower leg should prompt further investigation to prevent the morbidity associated with unnecessary surgery.

## Introduction

Cutaneous squamous cell carcinoma (cSCC) has several clinical and histopathologic mimics that can present a diagnostic challenge [1]. Not all histologically diagnosed SCCs should be treated with surgery, and clinical pathologic correlation is essential when making a diagnosis and developing a treatment plan. We present seven patients referred to Mohs Clinic for treatment of cSCC for whom multiple surgeries were avoided by assessing the clinical situation, recognizing an alternative diagnosis, and prescribing appropriate therapy for the underlying condition. 

## Materials and methods

We present a case series of seven female patients presenting to a single institution for treatment of biopsy-proven cSCC of the lower leg. A retrospective chart review was performed, and patient demographics, case details, and clinical and histological images were collected from the electronic medical record (Epic). 

## Results

Case 1

A 48-year-old female with a history of chronic hepatitis C virus (HCV), anxiety disorder, and rectal carcinoma presented for treatment of multiple biopsy-proven superficially invasive cSCC. Examination revealed over fifty 1 to 3 cm excoriated scaly pink plaques on the forehead, neck, and bilateral upper extremities. The patient had multiple surgical scars over her extremities from prior excisional surgeries apparently for cSCC. Repeat biopsies at our institution supported the prior diagnosis of cSCC.

Upon further questioning, the patient revealed that pruritus and eroded lesions had developed approximately one year prior while undergoing capecitabine treatment for rectal carcinoma. To evaluate for a systemic cause of her symptoms, the patient was referred to hepatology and subsequently initiated on peginterferon alfa-2a and ribavirin for chronic HCV. Her symptomatic lesions were treated with oral doxepin (starting dose 10 mg nightly, titrated up to 150 mg), intralesional triamcinolone (20 mg/mL), and flurandrenolide tape. Repeat biopsies several months later revealed epidermal hyperplasia over dermal scarring, with no evidence of cSCC. All sites and pruritus were resolved at follow-up several years later.

Case 2

**Figure 1:**
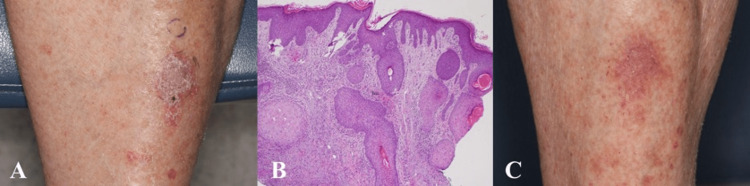
Representative images of Case 2 depicting the biopsied lesion (A) clinically at initial presentation, (B) histologically (4× magnification), and (C) clinically after treatment with flurandrenolide tape.

An 84-year-old female was referred to the Mohs surgery clinic for evaluation of several lesions on her lower extremities, clinically suspicious for cSCC. These had been present for several weeks and were pruritic. Of note, at the time of referral, she was also experiencing a generalized rash thought to be a lichen planus-like eruption as well as thrombocytopenia. Her past medical history was significant for prior cSCCs on her nose and temple treated with Mohs surgery. She also reported problems with gastroesophageal reflux and anxiety. She recently changed her proton pump inhibitor therapy from pantoprazole to omeprazole. Biopsies were performed of representative lesions on her hands to evaluate her generalized rash, and an additional sample was taken from a lower extremity lesion for which the patient was referred. Pathology of the hand lesions revealed a lichenoid and spongiotic dermatitis with rare eosinophils, while analysis of the lower extremity lesion was consistent with an invasive well-differentiated cSCC extending to all margins (Figure 1). Concluding that the patient may have lichen planus as a side effect from her proton pump inhibitor, she was instructed to discontinue omeprazole and apply flurandrenolide tape to the lower extremity lesions daily. One month later, her symptoms and lesions were much improved, and a re-biopsy of the site on her lower extremity revealed scar only. 

Case 3

A 79-year-old female with history of three keratoacanthomas excised over the past year presented for treatment of biopsy-proven cSCC and keratoacanthoma of her left leg and left arm, respectively. Both lesions had developed over the several months. Systems review revealed generalized pruritus over the previous six months, and extensive excoriations were apparent over the trunk and extremities.

Suspecting an inflammatory etiology, surgery was deferred and the patient was treated with oral doxepin 10 mg nightly and flurandrenolide tape daily. At follow-up, pruritus was significantly improved and the residual papules at both biopsy sites were completely resolved. A re-biopsy of both areas confirmed the absence of malignancy. Of note, the patient reported replacing the covering on her recliner with additional improvement in her symptoms, which she believed contributed to improvement. Several months later, she was referred to our institution again with a biopsy-proven cSCC of the right pretibial lesion. Surgery was deferred, and as her insurance would no longer cover flurandrenolide tape, she was instructed to apply clobetasol propionate under occlusion to the area. Reevaluation a month later showed flattening of the lesion and repeat biopsy at the site was negative for tumor.

Case 4

A 67-year-old female with a history of BCC presented for treatment of a biopsy-proven keratoacanthoma on her left shin. She stated that this and several adjacent lesions had developed over one year and were intensely pruritic. Examination revealed several firm pink excoriated papules on the left lower extremity and bilateral lower extremity edema. Surgery was deferred, and the patient was instructed to apply flurandrenolide tape daily. Within two months, all lesions had essentially resolved. Repeat biopsy revealed scar without evidence of cSCC.

Case 5

A 76-year-old female presented for evaluation of several pink scaly papules on her bilateral legs, clinically concerning for cSCC. Several weeks prior at an outside institution, she had two similar-appearing lesions on her left leg biopsied, diagnosed as well-differentiated cSCC, and treated with electrodessication and curettage. She described these lesions as *waxing and waning* and associated with constant pruritus. Upon further questioning, the onset of her symptoms corresponded to when she started furosemide for hypertension. Three clinically suspicious lesions were biopsied: one was reported at superficially invasive cSCC, the second as hypertrophic actinic keratosis (AK), and the third as scar.

Given the clinical context and temporal association with furosemide, a drug eruption was favored as a cause of the lesions and surgery was deferred. Desoximetasone 0.25% cream was prescribed daily, with resolution of the lesions within a few weeks.

Case 6

**Figure 2:**
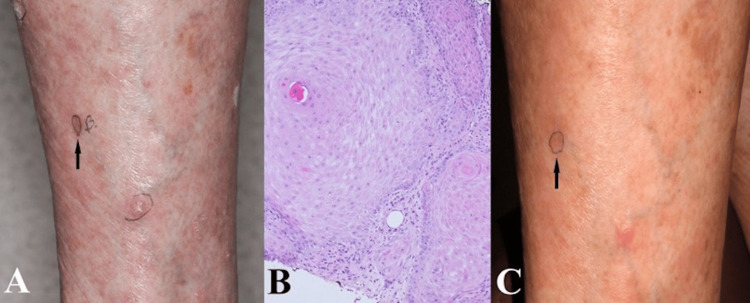
Representative images of Case 6 depicting the biopsied lesion (A) clinically at initial presentation, (B) histologically (10× magnification), and (C) clinically after treatment with doxepin and flurandrenolide tape.

A 74-year-old female with history of multiple non-melanoma skin cancers (NMSC), depression, and anxiety disorder unresponsive to sertraline presented for treatment of two biopsy-proven invasive cSCCs of her right lower leg. Examination revealed residual papules at the two biopsy sites in addition to numerous crusted papules and nodules of the bilateral distal lower extremities (Figure 2). The patient reported that four months prior, she treated AKs in the same area with 5-fluorouracil (5%) cream daily for one week. She subsequently developed a diffuse pruritic rash that was resistant to treatment with topical corticosteroids, oral prednisone, and hydroxyzine. Surgery was deferred, and the patient was diagnosed with allergic contact dermatitis. In collaboration with her primary care physician, doxepin was initiated to address both her pruritus and underlying mood disorder. She was instructed to apply flurandrenolide tape, which was later changed to clobetasol 0.05% cream due to expense. Within two months, her lesions and symptoms had resolved, and repeat biopsies revealed scar and stasis dermatitis.

Case 7

**Figure 3:**
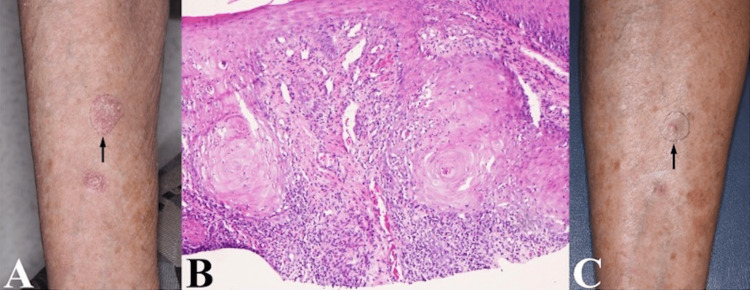
Representative images of Case 7 depicting the biopsied lesion (A) clinically at initial presentation, (B) histologically (10× magnification), and (C) clinically after treatment with doxepin and flurandrenolide tape.

A 78-year-old female was referred to Mohs Clinic for management of a recurrent cSCC on her leg. This had been excised 18 months prior, and when it recurred, she had received radiation to the area. Despite these measures, the lesion recurred again, was treated with standard excision, noted to have positive margins, and then re-excised and grafted. Following these interventions, new lesions had appeared around the margins of the operative site. Biopsies of these lesions were determined to be superficially invasive cSCC (Figure 3), which prompted her referral for further management. The patient reported experiencing pruritic lesions that caused nighttime discomfort. Additionally, she was receiving treatment with furosemide for lower extremity edema.

Examination revealed several pink keratotic papules and plaques on the lower extremities, the largest measuring 2.5 cm in diameter. Given her history of chronic lower extremity edema and diffuse lesions on examination, surgery was deferred. Treatment was initiated with flurandrenolide tape, oral doxepin, and application of an Unna boot on the side of the *recurrent* lesion. Two months later, all lesions had essentially resolved, and repeat biopsy at the original site was negative for cSCC.

## Discussion

Several diagnoses share histopathologic features with cSCC, including benign reactive conditions and other cutaneous malignancies. Therefore, an accurate diagnosis is essential to prevent unnecessary surgery for patients [1,2]. While immunohistochemistry can help differentiate cSCC from non-squamous etiologies, it is difficult to distinguish between conditions associated with squamous proliferation. This includes pseudoepitheliomatous hyperplasia (PEH) - a reactive proliferation of the epidermis that develops secondary to numerous infectious, inflammatory, and neoplastic etiologies [3]. Conditions associated with PEH can also mimic cSCC clinically, confusing diagnosis and treatment [4]. This diagnostic dilemma has led to attempts to objectively differentiate between PEH and cSCC. Using a polymerase chain reaction (PCR) assay, Su et al. found that PEH and cSCC inversely express C15orf48 and KRT9, two highly discriminatory genes [5].

The patients in this series had lesions secondary to a wide variety of etiologies that mimic cSCC (Table 1). Case 2 patient was diagnosed with drug-induced hypertrophic LP, a condition that can present histologically with PEH and is difficult to differentiate from cSCC clinically in some cases [6]. The other cases represent lesions related to generalized pruritus or an underlying inflammatory condition, namely, hepatitis C, contact dermatitis, and stasis dermatitis. Case 1 patient developed pruritic lesions in the context of chronic HCV, and her symptoms improved after combining symptomatic treatment with antiviral therapy. Of note, chemotherapy can lead to the reactivation of chronic HCV, which may explain why her symptoms initially developed shortly after beginning capecitabine for rectal carcinoma [7]. Two patients were diagnosed with ACD, which can also demonstrate PEH on histology [8]. Case 3 patient reported improvement in symptoms after replacing the fabric covering on her recliner. The likely sensitizer was dimethyl fumarate, an antifungal agent identified as a cause of ACD from furniture manufactured in China [9]. Case 6 patient developed a diffuse rash shortly after applying topical 5-fluorouracil, which is also a known trigger of ACD [10].

**Table 1:** Patient characteristics and treatment details, separated by final diagnosis. Pt, patient; F, female; cSCC, cutaneous squamous cell carcinoma; OTS, occluded topical steroids; ILS, intralesional steroids; TS, topical steroids; LP, lichen planus; PN, prurigo nodularis; a/w: associated with; HCV, hepatitis C virus; ACD, allergic contact dermatitis

Pt	Age/Sex	Location	Pruritus	Initial biopsy	Treatment			Etiology	Repeat biopsy	Follow-up without new cSCC (years)
					Doxepin	OTS	Other			
1	48/F	H/UE	×	cSCC	×	×	ILS	Chronic HCV	Scar	9
2	84/F	UE/LE	×	cSCC		×		Hypertrophic LP	Scar	5
3	79/F	UE/LE	×	cSCC		×		ACD	Scar	4
4	67/F	LE	×	cSCC		×		Stasis dermatitis	Scar	6
5	76/F	LE	×	cSCC			TS	Drug eruption	Scar	5
6	74/F	LE	×	cSCC	×	×		ACD	Scar	1
7	78/F	LE	×	cSCC	×	×		Stasis dermatitis	Scar	0.75

Therapeutic trials involve several approaches, including discontinuing potentially causative medications, avoiding potential allergens, adopting improved skin care regimens, and utilizing topical or intralesional corticosteroids. The application of high-potency topical corticosteroids under occlusion enhances medication activity and acts as a physical barrier to prevent the traumatization of pruritic lesions [11,12]. The majority of our patients were treated with flurandrenolide tape, while one patient underwent treatment with high-potency topical steroids under an Unna boot. Additionally, another patient utilized a combination of high-potency steroids and tape due to insurance-related considerations. When stasis dermatitis is suspected, the use of compression and elevation is often beneficial [13].

In our experience, we have found doxepin, a tricyclic antidepressant, and strong H1 antagonist, to be a highly effective antipruritic agent in these patients, perhaps as it treats both histamine effects and underlying mood disorders [14,15]. Doxepin therapy should be initiated at a low dose and titrated to the patient’s response, and involving the patient’s primary care physician is often helpful in this regard. It is important to establish a close follow-up during therapeutic trials to gauge a response, proceeding with surgical management if the target lesions worsen or fail to improve.

When evaluating patients with cSCC, signs that may warrant further investigation include lack of significant photodamage, no prior skin cancer history, associated pruritus, and location on an extremity (often below the knee). These were common presenting features in our patients that guided us away from surgery. Supporting our diagnoses, our patients were monitored for an average of five years, during which no new cases of cSCC were observed (Table 1), and none of the patients returned during the follow-up period.

## Conclusions

Clinicopathologic correlation is essential in the presurgical evaluation of patients presenting with cSCC accompanied by pruritis. This case series highlights the occurrence of pruritic nodules generally below the knee in older women, which pathologically resemble cSCC but are attributable to a variety of alternative etiologies. Our experience suggests these lesions may also develop in the setting of concurrent episodes of stress, anxiety, or depression. We suggest this entity may not be as rare as the literature indicates. Recognizing this scenario will spare the patient unnecessary surgery and address an underlying dermatosis that may have gone undiagnosed for years.
